# How social support shapes entrepreneurial intentions: sequential gains in self-efficacy and attitude

**DOI:** 10.3389/fpsyg.2026.1764921

**Published:** 2026-03-10

**Authors:** Sichao Gao, Guoguo Zhao

**Affiliations:** Changzhou Vocational Institute of Textile and Garment, Changzhou, China

**Keywords:** chain mediation, entrepreneurial attitude, entrepreneurial intention, entrepreneurial self-efficacy, social support, university students

## Abstract

The aim of the study is to investigate the relationship between entrepreneurial intention, entrepreneurial self-efficacy, entrepreneurial attitude, and social support. The study also seeks to investigate the mediating impact of entrepreneurial self-efficacy (ESE) and entrepreneurial attitude (EA) on the relationship between entrepreneurial intention (EI) and social support (SS). Five hundred and thirty-eight Chinese college students were randomly selected and answered a questionnaire. The data was analyzed using linear regression and bootstrap. Results indicated that social support predicted self-efficacy (β = 0.783) and directly affected intention (β = 0.198). Self-efficacy (β = 0.813) and attitude (β = 0.636) predicted intention. Self-efficacy promoted attitude (β = 0.168) was also found. Furthermore, the chain mediation SS → ESE → EA → EI was significant. Finally, the model explained 77.8% of the variance of intention. In summary, social support helps facilitate entrepreneurial intention through the method of “Self-efficacy → attitude”, showing that universities strengthen support networks and ability experiences to transform entrepreneurial intention.

## 1 Introduction

Entrepreneurial intention (EI) is considered a proximal antecedent of entrepreneurial behavior, thus serving as an important psychological gateway for explaining the individual's venture creation behavior. Among the foremost frameworks that rely on intention, the theory of planned behavior (TPB) argues that intention is determined both by the attitude toward the behavior as well as the perceived social pressure and perceived behavioral control. TPB thus provides a parsimonious yet explanatory account of how motivational forces are translated into planned action (Ajzen, 1991). Entrepreneurship is a process leading to the creation of an organization and is distinguished from management of established organization. Entrepreneur refers to the individual who undertakes the risk of setting up and managing a firm for commercial purpose. Yet, the challenge persists that intention does not guarantee action. This underscores the importance of identifying pooled credentials that not only foster the formation of intention but also facilitate its translation into subsequent behavior (Sheeran, 2002; Kautonen et al., 2015).

One of such contextual resource is social support. According to the stress and coping literature, social support is proposed to ameliorate strain and to furnish information and emotional and instrumental resources that enhance coping and goal pursuit (Cohen and Wills, 1985). Social support is defined as “the perception and actuality that one is cared for, has assistance available from others, and is part of a supportive network.” If we apply this definition to entrepreneurship (the founders' perspective), we are talking about relational capital. Social support informs, encourages, and offers concrete assistance, thereby enhancing the perceived feasibility and lowering the perceived challenge of starting a business. This contextual resource perspective is comparable to social cognitive accounts in which environments help shape self-regulatory beliefs and motivate goal-directed behaviors (Blumenthal et al., 1987; Lent et al., 1994).

Entrepreneurial self-efficacy may be the mechanism through which social support operates—individuals' beliefs about their capability to successfully perform entrepreneurial tasks. Self-efficacy, which is a major building block of social cognitive theory, is shaped by things in one's environment, like social persuasion, vicarious learning, and mastery experiences (Bandura, 1977). In entrepreneurship contexts, ESE has the potential to distinguish entrepreneurs from managers and predict entrepreneurial outcomes. By injecting some theory, we proposed ESE as a meaningful pathway linking social context to entrepreneurial motivation (Chen et al., 1998; Hao et al., 2005). Another important mechanism is the entrepreneurial attitude (EA), which represents the overall positive or negative evaluation of individuals on becoming an entrepreneur; according to the TPB, attitude is a direct antecedent of intention and an important evaluative step in the intention formation process (Ajzen, 1991; Schlaegel and Koenig, 2014). Conceptually, ESE may influence EA by elevating perceived feasibility and anticipated success, rendering entrepreneurship to appear more attractive and worthwhile and thereby strengthening EI (Lent et al., 1994; Hao et al., 2005).

Although these theoretical links exist, existing empirical work has often looked at the roles of ESE and EA as parallel mediators or as psychological correlates of EI rather than as a sequential process. In other words, social cognitive accounts and the Theory of Planned Behavior (TPB) imply a “capability belief → evaluative stance → intention” chain; this has not been consistently modeled as a serial mechanism through which social support influences Emotional Intelligence (EI) (Ajzen, 1991; Lent et al., 1994). Tackling the identified gap is important as one sequential model outlines the process through which supportive social environment first build capability beliefs (ESE) which generate evaluative judgments (EA) which cause the EI explaining the formation of intention in a more process-consistent way than mediation models (Hao et al., 2005; Schlaegel and Koenig, 2014).

It is pertinent to this question considering the Chinese university context. Cultural models of the self-highlight that East Asian contexts foster interdependent self-construal's, whereby individuals are more attentive to relational expectations and social embeddedness in their choices (Markus and Kitayama, 1991; Cross et al., 2002). According to Bian (2018), relationship and guanxi networks have the capability to significantly shape access to information, resources and opportunities in China. This, in turn, can heighten the motivational salience of perceived support for forming career intentions like entrepreneurship. Furthermore, Chinese data suggest that university and environmental support can influence students' EI through ESE and beliefs about-related attitudes, indicating tests should be context sensitive (Hou et al., 2019; Lu et al., 2021).

Consequently, this study seeks to ascertain whether social support predicts emotional intelligence both directly and indirectly via the various interventions of self-efficacy and emotional approach. Also, via an intervention pathway where social support enhances self-efficacy which enhances emotional approach and thus emotional intelligence. This theoretical model links social support as a context resource with a sequential psychological process to further clarify how supportive environments develop entrepreneurial motivation of Chinese university students. According to (Liñán and Chen 2009), the evaluation of EI uses established instrumentation widely across cultures.

## 2 Literature review

### 2.1 Social support and entrepreneurial intention

Social support broadly refers to the emotional, informational and instrumental resources they perceive from family, friends, teachers, the school and other institutions surrounding the individual (Blumenthal et al., 1987). Within entrepreneurship research, social support is recognized as an important contextual resource at the entrepreneur's disposal that can reduce uncertainty and perceived risks, facilitate access to material and information assets, and increase psychological preparedness for becoming an entrepreneur. Consequently, it is an essential antecedent of EI, especially in the case of university students and young adults who, in the context of venture evaluation and pursuit, mostly turn to social networks.

Existing literature shows a positive association between perceived social support and emotional intelligence (EI). An example of the study of Yesmin et al. (2024) shows that educational and social support indirectly impact EI via psychological constructs from TPB. The experience of social support has been found to not only directly predict emotional intelligence or EI but also influence how someone perceives one's own traits, identity, and what one can do. This claim is backed by similar findings in various cultural and educational contexts. According to Wang et al. (2023), perceived social support acts as a partial mediator in the relationship between extraversion and EI, while Ratkovic et al. (2025) present evidence that it might act as a moderator to the relationship between diversity-related characteristics and EI.

Social support emerges as a particularly impactful influencer to both Chinese and international students. Students' confidence in their own abilities would become more established as the support networks increase, thereby causing perceived entrepreneurial risks to lessen and subjective norms to strengthen. For instance, Qin et al. (2024) revealed that social support mediates relationship between psychological traits and EI among Chinese students in South Korea. Zhang et al. (2022) demonstrated that social status and proactive personality interacts with supportive systems to shape entrepreneurial career intentions. Such findings highlight perceived social support as an important contextual determinant of EI among students.

Though, most research looks at the direct effects of social support or how social support works as a single mediator or moderator. Numerous university students have been significantly affected by various emotional issues resulting from the pandemic. The availability of university health and counseling services (UHS), along with other forms of virtual support, has been crucial during this time. This situation underscores the necessity of developing models that link social support to entrepreneurial cognition and motivation through sequential processes.

### 2.2 Entrepreneurial self-efficacy as a predictor of entrepreneurial intention

Entrepreneurial self-efficacy (ESE) refers to a person's beliefs about their capabilities of performing various entrepreneurial tasks like risk management, opportunity recognition and resource coordination (Boyd and Vozikis, 1994). The SCT foundation of ESE refers to a motivational construct that affects people's choice to start, continue, or develop their entrepreneurial activity. It affects the degree and scale of engagement.

Many researchers consistently show that ESE and EI are positively related. The seminal research by Boyd and Vozikis (1994), followed by Chen et al. (1998) shows that people with higher ESE are more likely to see entrepreneurship as a career option. And, Hsu et al. (2019) and Simba et al. (2025) found that people with more ESE show more willingness to search, and shake up and keep going (Chen et al., 1998; Lucas and Cooper, 2004; Hsu et al., 2019; Simba et al., 2025). As a whole, the findings qualify ESE the most stable cognitive predictor of EI.

As per SCT, the development of self-efficacy is influenced by mastery experience, vicarious learning, verbal persuasion, emotional regulation, and social-support systems (Bandura, 1977). The assistance of family, friends, mentors and institutions through positive reinforcement and guidance enhances the way one perceives his/her capabilities. This making them feel that a challenge is something to be played with, not a threat. Support for thesis is empirical. Specifically, Hsu et al. (2019) demonstrate that entrepreneurship fit interacts with entrepreneurial self-efficacy (ESE) to shape entrepreneurial intention (EI). Further, Hao et al. (2005) show that entrepreneurial experience and risk propensity influence intent mainly through ESE.

Nevertheless, due to the aforementioned discoveries, the majority of the existing research has regarded ESE in isolation as either a single mediator or even a single predictor while hardly looking at the interaction of ESE with other cognition-based variables, particularly EA. This limitation constrains the understanding of the sophisticated psychological mechanisms through which contextual factors such as social support are internalized and ultimately transformed into EI.

### 2.3 Entrepreneurial attitude as a determinant of entrepreneurial intention

EA is the general evaluation of an individual with regard to entrepreneurship as a good and worthwhile career option. Based on the TPB (Ajzen, 1991), EA is one of the most powerful proximal predictors of EI as it measures the extent to which an individual considers initiating a starting venture attractive, worthwhile and meaningful.

Research consistently shows that more positive EAs are associated with stronger EIs. The personal attributes, previous exposure to entrepreneurship, opportunity recognition as well as the perceived contextual support are factors affecting EA. For instance, Jena (2020) says that students' evaluations of entrepreneurship education being positive boosts students' EIs. Likewise, Galvão et al. (2024) highlight that positive EAs being developed through support systems in universities boosts students' EIs.

While EA is a well-known quasi-synonymous suggestion of EI, what is the importance of EA in more complex psychological sequences? Only a few studies have analyzed EA as the result of ESE and as the proximal predictor of an intention within the sequential mechanism. People having a high ESE tend to see more control and less risk in entrepreneurial tasks which can engender more positive EAs and, by extension, more robust EIs. The above sequence awareness is required to understand how cognitive evaluation relates perceived ability to attitude and that attitude to EI.

### 2.4 Toward a unified chain mediation model of entrepreneurial intention

Recent studies have shown that EI is hardly the function of one cognitive antecedent. Rather it is a product of many psychological processes (perceived capability, affective evaluation, normative) working together. Nevertheless, far too many empirical studies examined mediators like ESE or EA in isolation, leaving only a partial understanding of how contextual resources are internalized and transformed into entrepreneurial motivation.

According to theory, SCT and the TPB equally support that social support affects ESE which affects EA which affects EI. Social support can improve ESE by providing supportive feedback, informational input, and vicarious learning. More intensive enhancement of self-efficacy can enhance coping appraisal by diminishing perceived barriers. Positive EAs can enhance the EI further. This pathway which holds SS → ESE → EA → EI, integrates contextual factors and psychological factors into one coherent explanatory system effectively.

Nonetheless, little empirical evidence has been generated in regards to this sequential process, particularly in the case of Chinese university students. People in this population generally have comparatively limited experience of real-world work and entrepreneurship with higher reliance on external support systems and a greater sensitivity to uncertainty in career decision-making These traits suggest that social support is the starting point of a psychological process that succeeds one another in enhancing ESE and EA that finally affects EI greatly. However, the integrated chain mediation models concerning this process are relatively scarce in the context of Chinese higher education.

Drawing from the theoretical foundations and empirical gaps stated above, the paper proposes a complete chain mediation model in which ESE and EA are both independent and sequential mediators between social support and EI. Social support is theorized to positively correlate with ESE, EA and EI, and that indirect effects will be generated through multiple mediation channels including ESE and EA. Based on this framework, the following hypotheses have been formulated.

H1: Social support has a positive effect on ESE.
H2: Social support has a positive effect on EA.
H3: Social support has a positive effect on EI.
H4: ESE has a positive effect on EI.
H5: EA is a positive predictor of EI.
H6: ESE has a positive effect on EA.
H7: ESE mediates the effect of social support on EA.
H8: EA mediates the effect of ESE on EI.
H9: ESE mediates the effect of social support on EI.
H10: EA mediates the effect of social support on EI.

Evidence from our research demonstrates that H11 is significantly true and has veracity and accuracy.

## 3 Methods

### 3.1 Participants and sampling procedure

The research participants were a sample of undergraduate students from universities and vocational institutions in Jiangsu province, China. The sampling design was convenience sampling, which was chosen because of easy access to student populations and restrictions imposed on large surveys. According to Department of Education of Jiangsu Province (2025), there are about 1.2 million higher-vocational students in Jiangsu province, and based on Wu (2018), the minimum size of the sample sufficient to perform the statistical analysis is 384, according to the following formula *N* > 100 000; *p* = 0.50; α = 0.05; *z* = 1.96. The science and Technology Division of the Changzhou Vocational Institute of Textile and Garment approved the survey in accordance with the Declaration of Helsinki.

**Figure 1:**
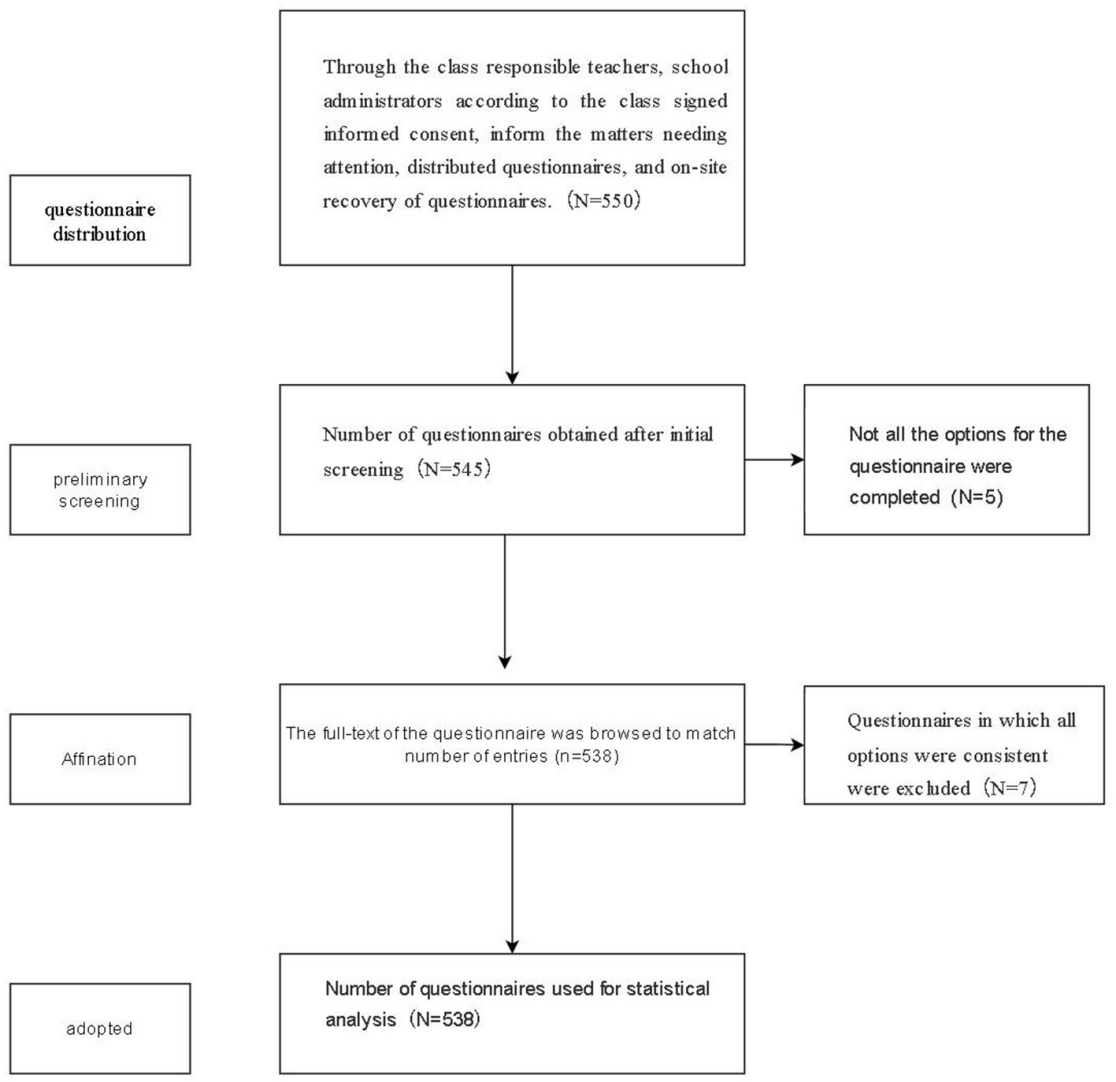
Flow chart of questionnaire data acquisition and processing.

To secure the necessary statistical power of sufficient sample size for SEM, Wenjuanxing, a commonly used online questionnaire platform in China, was used to administer electronic questionnaires in the administrative class. Out of a total of 550 responses collected, a final total of 538 valid questionnaires were retained (Figure 1) after removing 12 invalid questionnaires with patterned or missing answers. Hence, a valid response rate of 97.81%.

Sole final sample consisted of 250 male (46.46%) and 288 female respondents (53.54%) whose mean age was 19.35 (SD = 1.25). The composition of the participants in terms of academic year was freshman 22.86%, sophomore 16.35%, junior 22.32%, and senior 38.47%. As per their disciplinary background, 51.3% and 48.7% were in science and humanities programs, respectively. Furthermore, 10.6% of the respondents had past experiences in entrepreneurship.

### 3.2 Measures

The scales were developed in Chinese and previously used by other studies. Each of the items was rated on a five-point Likert-type scale ranging from 1 = strongly disagree to 5 = strongly agree. Thus, higher scores reflected a higher level of the corresponding construct.

#### 3.2.1 Entrepreneurial intention

The EI Scale developed by Liang and Yi (2016) was used. It has eight items and two dimensions, a career-pursuit EI and career-alternative EI. In this research, the original scale is used which has good psychometric properties and it exhibited excellent internal reliability (Cronbach's α = 0.958). The CFA results confirmed the construct validity of the J-Y matrix. GFI = 0.977, AGFI = 0.932, CFI = 0.993, TLI = 0.983, RMSEA= 0.077.

#### 3.2.2 Entrepreneurial self-efficacy

We utilized Tang's (2009) ESE scale given the multi-construct characteristics of the survey and our SEM framework. In this research, one representative item was chosen from each of the five venture-exploration task domains (innovation efficacy, risk-taking, opportunity identification, relationship coordination, and goal commitment) so that ESE is modeled as a latent construct with five reflective indicators capturing its conceptual breadth while maintaining an efficient measurement model. This strategy aligns with guidelines for constructing parsimonious indicators in structural equation modeling. Furthermore, these indicators can be an appropriate choice if coverage across facets on content is preserved. The index demonstrated excellent internal consistency (α = 0.968) as well as an acceptable fit in a CFA (GFI = 0.948, AGFI = 0.906, CFI = 0.987, TLI = 0.980, RMSEA = 0.066).

#### 3.2.3 Entrepreneurial attitude

Fang's (2017) EA Scale, which captures entrepreneurship evaluative judgements of students, was used to assess EA. Items were scored on a five-point format, whereby higher scores signified more positive responses. According to alpha 0.944, the reliability of the scale is high. The CFA also indicated a satisfactory fit of the model with GFI= 0.989, AGFI = 0.958, CFI = 0.996, TLI = 0.991 and RMSEA = 0.072.

#### 3.2.4 Social support

Social support was assessed with a multiple scale based on research (Blumenthal et al., 1987). There is a scale which is translated into Chinese by Jiang G et al. that measures emotional, informational and instrumental support from family, peers and teachers (Huang et al., 1996). The scale showed good reliability, and confirmatory factor analysis (CFA) affirmed its satisfactory psychometric quality.

### 3.3 Procedure

The course instructors administered the online survey in classroom settings to collect the data. Respondent's voluntary participation was anonymous and electronic informed consent was administered to them before filling in the questionnaire. In line with the normative ethical conditions, identifying information was not collected.

#### 3.3.1 Data analysis strategy

Data analysis was done in SPSS 27.0 and AMOS 28.0 in two stages. To begin, descriptive statistics and *t*-tests/ANOVAs were conducted to investigate potential differences in study variables across gender, grade, major, birthplace and entrepreneurial experience. To investigate the common method variance, all self-reported variables were subjected to Harman's one-factor test. A principal component analysis (without rotation) showed that the first factor accounted for 32.34% of the variance and is less than 40%. This implies that common method bias is not serious. All variables were analyzed for means, SDs, and Pearson correlations. In a second step, the SEM using maximum likelihood estimation was employed to test direct, independent, sequential, and total effects. The data fit was assessed in accordance with well-established criteria, including CMIN/DF, GFI, AGFI, NFI, TLI, CFI, RMSEA, and RMR. The estimations of indirect effects through multi-mediation were tested with the SEM techniques with squared multiple correlations (*R*^2^) for endogenous variables. *R*^2^ tells us the proportion of variance explained by our model for the endogenous variable.

## 4 Results

### 4.1 Common method bias test

Because all the measures were entirely self-reported data a Harman's single-factor test was conducted to test common method bias. Findings from the unrotated principal component analysis (PCA) indicated that 12 factors with eigenvalues > 1. First factor accounts for 32.34% of total variance. This is lower than 40% threshold (Zhou and Long, 2004). These results suggest that the common method bias was unlikely to pose a serious threat to the results.

### 4.2 Overall descriptive statistics

As indicated by Table 1, descriptive statistics for the four main variables show that students reported moderately high perceptions of social support (*M* = 3.67), EA (*M* = 3.79), and ESE (*M* = 3.54). Conversely, the EI's mean was lower (*M* = 3.34) which means that these students feel that they have the support from their living environment and that they have a positive attitude and confidence toward entrepreneurship, but they are less willing to convert their perceptions into firm EIs.

**Table 1:** Overall descriptive statistics of the key variables.

**Variable**	**Mean**	**SD**	**Min**	**Max**
SS	3.669	0.931	1.00	5.00
EA	3.785	0.946	1.00	5.00
ESE	3.538	0.889	1.00	5.00
EI	3.342	1.042	1.00	5.00

*Note* All variables are reported as mean scores on a 1–5 Likert scale (converted from total scale scores). SS, social support; ESE, entrepreneurial self-efficacy; EA, entrepreneurial attitude; EI, entrepreneurial intention.

This matching pattern found here is consistent with earlier studies showing that the conversion from entrepreneurial preference or self-belief to firm EI is more often curtailed by perceived risks, resource constrains, and broader contextual uncertainties than actual resource shortage. The researchers examined differences across demographic groups using independent-samples t-tests and one-way ANOVA (Table 2), revealing several significant patterns. Female students reported significantly higher Emotional Intelligence than male students (*p* < 0.01); there were no significant gender-based differences in social support, EA, or ESE.

**Table 2:** Differences in key variables.

**Group variable**	**Comparison**	**Variable**	**Mean1**	**Mean_2_**	***t***	***p***
Gender	Male vs. Female	SS	3.625	3.707	1.026	0.305
Gender	Male vs. Female	EA	3.711	3.849	1.682	0.093
Gender	Male vs. Female	ESE	3.488	3.581	1.225	0.221
Gender	Male vs. Female	EI	3.216	3.451	2.619	0.009
Local	Urban vs. Rural	SS	3.753	3.594	1.973	0.049
Local	Urban vs. Rural	EA	3.779	3.790	0.137	0.891
Local	Urban vs. Rural	ESE	3.601	3.481	1.546	0.123
Local	Urban vs. Rural	EI	3.350	3.334	0.178	0.859
Entrepreneurial experience	Yes vs. No	SS	3.792	3.655	0.814	0.419
Entrepreneurial experience	Yes vs. No	EA	0.867	3.775	0.554	0.581
Entrepreneurial experience	Yes vs. No	ESE	3.772	3.510	1.692	0.096
Entrepreneurial experience	Yes vs. No	EI	3.721	3.297	2.637	0.010
Grade (ANOVA)	Year 1/2/3/4	SS	–	–	4.599	0.003
Grade (ANOVA)	Year 1/2/3/4	EA	–	–	1.626	0.182
Grade (ANOVA)	Year 1/2/3/4	ESE	–	–	1.852	0.137
Grade (ANOVA)	Year 1/2/3/4	EI	–	–	2.465	0.062

*Notes* Mean1 and Mean_2_ represent the mean scores for the two comparison groups; bold values denote statistically significant differences at *p* < 0.05, and ANOVA results are reported for Grade owing to its multi-group structure. SS, social support; ESE, entrepreneurial self-efficacy; EA, entrepreneurial attitude; EI, entrepreneurial intention.

When comparing urban and rural students, the former had significantly better social support perceptions. However, emotional attachment differences, emotional self-efficacy differences and emotional intelligence were not significant. According to the information provided in Table 2 of the article, students who had previous entrepreneurial experience, compared to those without, had higher levels of entrepreneurial intent (EI), slightly higher levels of entrepreneurial self-efficacy (ESE), and higher grade level was associated with more social support, and a marginal increase in EI.

The findings suggest that demographic characteristics result in different perceptions of support and entrepreneurial tendency by students. However, some of these characteristics are more independent than others.

### 4.3 Correlation analysis

Table 3 shows the Pearson correlation coefficients of the study variables. The correlation was positive and significantly different from zero at *p* < 0.01. Social support was strongly associated with ESE (*r* = 0.752) and EA (*r* = 0.746) and moderately associated with EI (*r* = 0.563). There was a high correlation between ESE and EA, *r* = 0.825 respectively and EI; *r* = 0.793. The relationships among the constructs are linear, and thus we can use SEM to examine the proposed mediation pathways. SEM was used to analyze the direct and indirect effects of the latent variables and the findings are presented in Table 4. Social support had ESE (β = 0.783, *p* < 0.001), EA (EA; β =0.284, *p* < 0.001), and EI (β = 0.198, *p* < 0.001), supporting H1–H3. ESE had a strong positive impact on EI (β = 0.813, *p* < 0.001) and EA (EA; β = 0.168, *p* < 0.001), supporting H4 and H6. EA positively predicted EI (β = 0.636, *p* < 0.001), supporting H5.

**Table 3:** Correlation coefficients among variables (*N* = 538).

	**SS**	**ESE**	**EA**	**EI**
SS	1			
ESE	0.752^**^	1		
EA	0.746^**^	0.825^**^	1	
EI	0.563^**^	0.793^**^	0.713^**^	1

^**^p < 0.01. SS, social support; ESE, entrepreneurial self-efficacy; EA, entrepreneurial attitude; EI, entrepreneurial intention.

**Table 4:** Path coefficients of the latent variables and hypothesis testing.

**Path**	**β**	***B***	**SE**	**CR**	***p***	**Hypothetical**
SS → ESE	0.783	0.762	0.038	20.217	^***^	H1
SS → EA	0.284	0.300	0.045	6.649	^***^	H2
SS → EI	0.198	0.209	0.055	3.773	0.011^*^	H3
ESE → EI	0.813	0.882	0.077	11.469	^***^	H4
ESE → EA	0.168	0.168	0.066	2.538	^***^	H5
EA → EI	0.636	0.692	0.049	14.078	^***^	H6

^*^p < 0.05, ^***^p < 0.001. SS, social support; ESE, entrepreneurial self-efficacy; EA, entrepreneurial attitude; EI, entrepreneurial intention.

The results obtained from the model fit indices indicate that this structural model fits well. The respective CMIN/DF = 2.137, GFI = 0.953, AGFI =0.968, NFI = 0.942, IFI = 0.953, CFI = 0.955, TLI = 0.945, RMSEA = 0.048, RMR = 0.035.

### 4.4 Mediating effect

**Figure 2:**
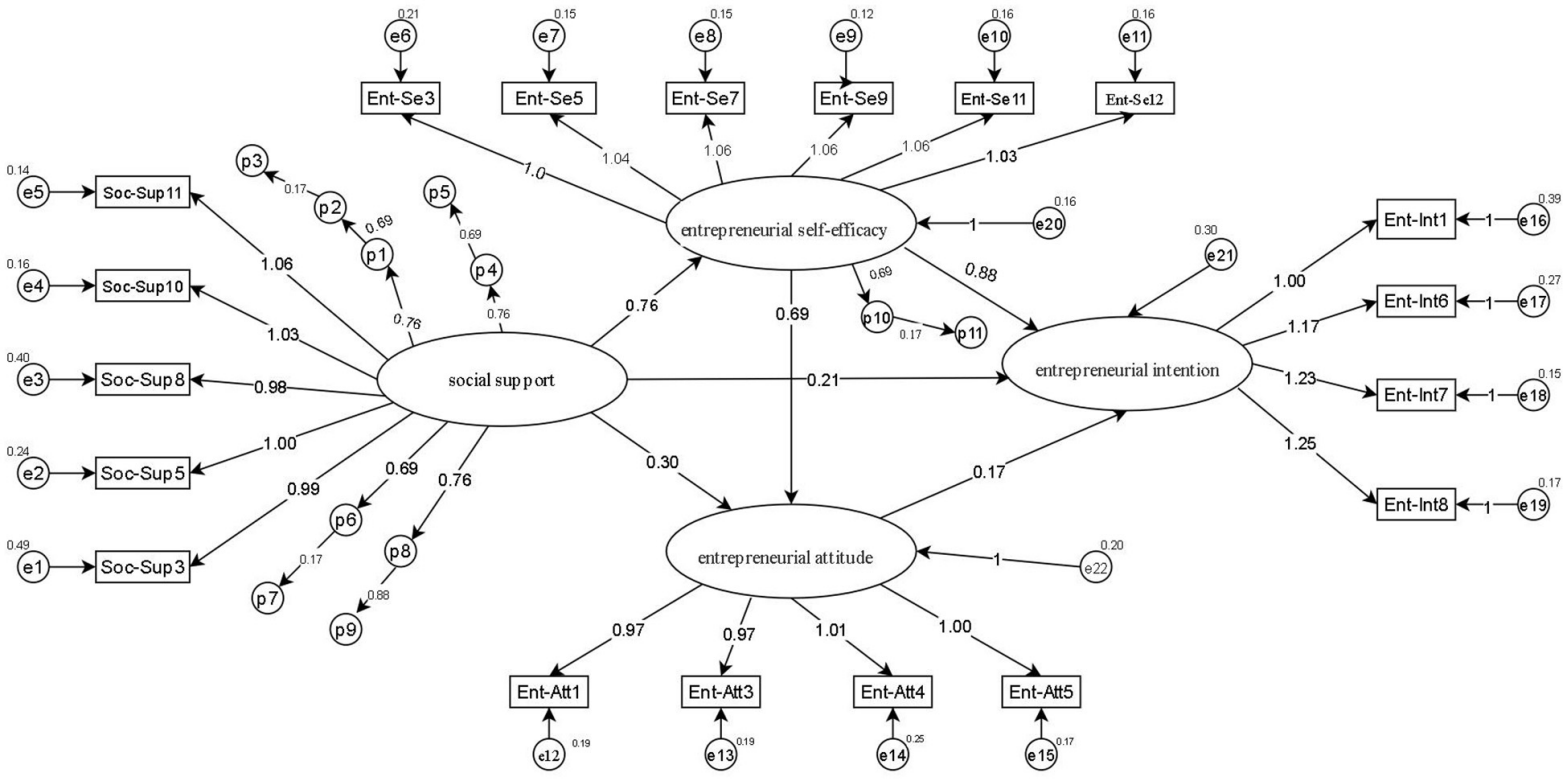
Non-standardized path coefficient plot of mediating effect.

In AMOS Phantom, a multiple mediation SEM (Figure 2) was tested, which revealed double mediation ESE and EA on the social support and EI relationship. Indirect effect analysis was used to estimate the dual mediating effects. The results of the mediating path analysis, which confirmed the findings using phantom variables, can be seen in Figure 2 and Table 5. Phantom modeling was performed using AMOS to estimate the specific indirect effects as shown in Table 5.

**Table 5:** Mediating effects among the variables.

	**Path**	**Coefficient of non-standardization**	**S.E**	***p***	**Standardized coefficients**
p5 (H7)	SS → ESE → EI	0.527	0.052	0.006^**^	0.631
p7 (H8)	ESE → EA → EI	0.116	0.060	0.001^***^	0.047
p9 (H9)	SS → ESE → EI	0.672	0.085	0.011^*^	0.499
p11 (H10)	SS → EA → EI	0.116	0.060	0.001^***^	0.951
p3 (H11)	SS → ESE → EA → EI	0.088	0.045	0.001^***^	0.108

^*^p < 0.05, ^**^p < 0.01, ^***^p < 0.001, SS, social support; ESE, entrepreneurial self-efficacy; EA, entrepreneurial attitude; EI, entrepreneurial intention.

The following are the indirect effects: social support → ESE → EI (β = 0.527, *p* < 0.001; H7 supported), social support → EA → EI (β = 0.116, *p* < 0.01; H8 supported), social support → ESE → EA (β = 0.672, *p* < 0.01; H9 supported), ESE → EA → EI (β = 0.116, *p* < 0.01; H10 supported).

We found support for H11 since the path, social support → ESE → EA → EI, was significant (β = 0.088, *p* < 0.01). The outcome indicates that social support influences emotional intelligence through independent and sequential psychological mediators. According to Table 6, the model fits well with *R*^2^ equal to 0.602 for ESE, 0.724 for EA and 0.778 for EI. The aforementioned figures imply that the integrated model explains a significant variance in EI among university students.

**Table 6:** Multiple squared correlations of latent variables.

**Variance**	***R* ^2^**
ESE	0.602
EA	0.724
EI	0.778

ESE, entrepreneurial self-efficacy; EA, entrepreneurial attitude; EI, entrepreneurial intention.

All of the 11 hypotheses (H1–H11) were supported (Table 7), providing solid empirical support for the theoretical model the authors built. Social support has both direct and indirect effect on EI through a combination of independent and serial mediation of ESE and EA.

**Table 7:** Hypothesis validation.

**Number**	**Hypothesis**	**Result**
H1	SS has a positive impact on ESE	Established
H2	SS has a positive impact on EA	Established
H3	SS has a positive impact on EI	Established
H4	ESE has a positive impact on EI	Established
H5	EA positively impacts EI	Established
H6	ESE has a positive impact on EA	Established
H7	ESE plays a mediating role in the relationship between SS and EI	Established
H8	EA plays a mediating role in the relationship between SS and EI	Established
H9	ESE mediates the relationship between SS and EA	Established
H10	EA mediates the relationship between ESE and EI	Established
H11	ESE and EA will play a dual intermediary role in the relationship between SS and EI	Established

SS, social support; ESE, entrepreneurial self-efficacy; EA, entrepreneurial attitude; EI, entrepreneurial intention.

## 5 Discussion

The present study seeks to explicate how social support influences entrepreneurial intention (EI) through a sequential psychological process that involves entrepreneurial self-efficacy (ESE) and entrepreneurial attitude (EA). Results showed that social support had a positive relationship with ESE (β = 0.783), EA (β = 0.284) and EI (β = 0.198, *p*= 0.011). Supportive environments are related to the funding of entrepreneurship concerning goal formation (Cohen and Wills, 1985). The strongest association with EI was ESE (β = 0.813) among direct predictors. EA also exhibited a positive, sizeable association with EI (β = 0.636). This is in line with intention-based models that highlight the centrality of capability beliefs as well as evaluative stance as crucial predictors of intention (Ajzen, 1991; Hao et al., 2005; Schlaegel and Koenig, 2014). The integrated model accounted for a substantial amount of variance (*R*^2^ = 0.778 for EI), indicating that it actually captures meaningful motivational dynamics.

Mediation analyses were able to furnish more specific process evidence. Social support has a significant indirect effect on EI through ESE (SS → ESE → EI; coefficient = 0.527, *p* = 0.006). This suggests that the presence of supportive networks may strengthen intention to a large extent by enhancing the perceived entrepreneurial capability of students. This finding is consistent with social cognitive theory in which social persuasion and resources availability lead to stronger efficacy beliefs that motivate challenging goal pursuit (Bandura, 1977). Social support also had an indirect effect on EA (SS → EA → EI); coefficient 0.116, *p*-value 0.001 which support a TPB-consistent elaboration that supportive social contexts can enhance individuals' evaluation of entrepreneurship thus increasing intention (Ajzen, 1991). Evidence of a serial mechanism was found, whereby social support predicted EA through ESE (SS → ESE → EA; coefficient = 0.672, *p* = 0.011). Furthermore, the serial indirect effect from social support to EI through ESE and EA was significant (SS → ESE → EA → EI; coefficient = 0.088, *p* = 0.001). Theoretical coherence of this serial pattern with a “capability belief evaluative stance intention” process parallels social cognitive accounts in which efficacy beliefs further down shape the evaluative cognitions and the expectancy-related cognitions giving rise to preferences and goal intentions (Lent et al., 1994; Hao et al., 2005).

The results also contribute to the contextual relevance of this mechanism in Chinese university students. As per the cultural theories, there is increased sensitivity to cues that communicate relationships and support (Markus and Kitayama, 1991; Cross et al., 2002). Furthermore, when support is perceived, it can have a significant impact on self-beliefs as well as attitudinal judgments. Also, the salience of guanxi networks in China implies that social embeddedness can shape perceptions of access to information and opportunities which can further reinforce this perceived support is important in motivating entrepreneurship as a viable pathway (Bian, 2018). Gaming in Chennai Findings from China point to university support impacting EI through ESE and attitudinal or TPB beliefs, which fits with the present pattern (Hou et al., 2019; Lu et al., 2021). Considering all the results, promoting entrepreneurship among Chinese students appears to be more effective not just by means of providing informational or instrumental support, but by targeting the psychological routes through which support leads to stronger efficacy and more pro-attitudes.

Basically, these findings indicate that universities and policy programs should adopt multi-component strategies. On the one hand, they need to offer structured social and mentoring support that enhances mastery experiences and social persuasion (which will strengthen ESE). On the other, they need to create experiential opportunities. Examples of experiential opportunities are project-based entrepreneurship courses, incubator participation, role-model exposure. As a result, heightened efficacy will lead to favorable entrepreneurial evaluations. Evidence suggests that the presence of university support can have an effect on EI in students through psychological beliefs including ESE and attitude-relevant perceptions (Lu et al., 2021). Furthermore, supports that exist after intention formation (i.e., access to networks, early-stage resources, continued mentoring etc.) may be required to mitigate the intention–action gap as intention is not always reflected in actions.

It should be noted that drawbacks exist. Due to the cross-sectional design that limits causal inference of the suggested sequence, longitudinal or experimental designs would be useful to more directly test temporal ordering. One likely limitation of our study is that self-reported data may lead to common method variance which may enhance associations. Future research should operationalize procedural remedies and multi-source indicators wherever possible. University students in China were the sample that was focused on. Although this is a good way to enhance contextual specificity, there is a need to replicate this across regions, educational systems, and non-students to evaluate the boundary conditions. Future research could examine whether the efficacy beliefs and attitudes are differentially strengthened by various types of social support (e.g., family, peer, institutional) and whether the serial mechanism predicts later entrepreneurial action under various resource constraints.

## 6 Conclusion

This research investigates the relationship between social support and EI of Chinese university students, as carried out by independent and sequential mediating roles of ESE and EA. According to the results of an integrated framework based on SCT and the TPB, social support is a critical contextual resource that affects EI either directly or indirectly.

To begin with, social support significantly contributes to ESE and EA, which highlights the importance of interpersonal and institutional environments for enhancing entrepreneurship confidence and evaluations. Furthermore, both ESE and attitude serve as essential mediating agents which seem to suggest students internalized the external supports through a cognitive–affective sequence that ultimately worked to strengthen EI. A comprehensive chain mediation model emerged from the validated study: social support → self-efficacy → attitude → intention. The social support and the other variables explained 77.8% of the variance in EI, which speaks for the theoretical significance of the model.

To sum up, the results suggested that students are more confident, have a positive evaluation of entrepreneurship and have stronger motivation toward entrepreneurship in a supportive social environment. As such, universities, educators, and policymakers should strategically foster social support networks and cultivate a diverse range of evidence-based entrepreneurship education practices to aid in students' entrepreneurial development.

This study offers great theoretical as well as practical insight into the sequential pathway in which social support influences EI for designing more effective entrepreneurship interventions which harbors the development of the next generation of innovative and resilient entrepreneurs.

## Data Availability

The data analyzed in this study is subject to the following licenses/restrictions: The original contributions presented in the study are included in the article/supplementary material, further inquiries can be directed to the corresponding author. Requests to access these datasets should be directed to ggzhao@cztgi.edu.cn.
